# Feeding infant formula with low sn-2 palmitate causes changes in newborn’s intestinal environments through an increase in fecal soaped palmitic acid

**DOI:** 10.1371/journal.pone.0324256

**Published:** 2025-05-28

**Authors:** Atsushi Ito, Hiromichi Shoji, Hiroko Arai, Satsuki Kakiuchi, Keigo Sato, Shinji Jinno, Naoto Takahashi, Kenichi Masumoto, Hitoshi Yoda, Toshiaki Shimizu

**Affiliations:** 1 Department of Pediatrics, The University of Tokyo Hospital, Hongo, Bunkyo-ku, Tokyo, Japan; 2 Department of Pediatrics and Adolescent Medicine, Juntendo University Graduate School of Medicine, Hongo, Bunkyo-ku, Tokyo, Japan; 3 Department of Neonatology, Toho University Faculty of Medicine, Omorinishi, Ota-ku, Tokyo, Japan; 4 Food Microbiology and Function Research Laboratory, Meiji Co., Ltd., Nanakuni, Hachioji, Tokyo, Japan; 5 Wellness Science Labs, Meiji Holdings Co., Ltd., Nanakuni, Hachioji, Tokyo, Japan; Mae Fah Luang University School of Anti Aging and Regenerative Medicine, THAILAND

## Abstract

**Background/Objectives:**

Soaped palmitic acid (PA) has been reported to be excreted in stool after feeding infant formulas containing low sn-2 palmitate levels, which corresponds to high sn-1 or -3 palmitate levels. While an *in vitro* study showed that soaped PA inhibits the *Bifidobacteria* growth, few clinical studies have evaluated effects of soaped PA on intestinal environments of infants. In this study, we aimed to evaluate associations between increased fecal soaped PA levels and inhibition of growth of the intestinal microbiome using clinical data, and to evaluate changes in the intestinal environment with formula-feeding.

**Methods:**

This study was conducted as a secondary analysis to our observational study of Japanese 1-month-old infants (n = 172). Infant formulas were classified into high sn-2 formula (≥ 50%) and low sn-2 formula (< 50%) according to the sn-2 binding ratio of PA. Multiple regression analyses and path analysis were performed as statistical analyses.

**Results:**

In the multiple regression analysis, the occupancy of *Bifidobacteria* was negatively correlated with the fecal soaped PA levels (β = −0.15, 95% confidence interval = −0.28– − 0.02). A path analysis suggested that low sn-2 formula feeding led to increased fecal soaped PA levels, decreased *Bifidobacteria* occupancy, and finally increased fecal pH.

**Conclusions:**

Our clinical data showed significant associations between higher fecal soaped PA levels and lower *Bifidobacteria* occupancy in the newborn gut, which agreed well with the report of the *in vitro* study. Our study also suggests that feeding infant formula with low sn-2 palmitate causes changes in the intestinal environment through an increase in fecal soaped palmitic acids.

## Introduction

Lipids are important energy sources, accounting for half the energy intake of infants. Palmitic acid (PA) is a saturated fatty acid, accounting for 20% of fatty acids and 10% of the energy sources in breast milk [1]. The absorption efficiency of PA varies based on its bonding position in triacylglycerol [2]. PA bound to the sn-2 position is not released by lipase and forms a monoacylglycerol. This aids in easy absorption because PA remains in a soluble form. In contrast, PA bound to the sn-1,3 position is released by lipase and becomes a free PA. The free PA binds to calcium in the intestine to form soaped PA, which is hard to absorb to the intestine due to its insolubility.

The sn-2 binding ratio of PA differs between breast milk and infant formula. While the sn-2 binding ratio of PA in breast milk is approximately 70% [2–6], that of infant formula is approximately 10% [5,7,8]. This is because vegetable oil blends, which are rich in sn-1,3 palmitate (with a binding ratio of approximately 90%), are commonly used as the main source of palmitic acid in infant formulas. Since high sn-1,3 palmitate lead to increased fecal soaped PA levels [9–13], a PA with a low sn-2 binding ratio may cause differences in intestinal environments between formula- and breast-fed infants.

The acidic intestinal environment of breast-fed infants is developed by the growth of *Bifidobacteria.* Breast milk contains high amounts of human milk oligosaccharides (HMOs), which are prebiotic components that help the growth of *Bifidobacteria* [14,15]. Higher growth rate of *Bifidobacteria* in the intestines lead to an acidic intestinal environment through the production of short-chain fatty acids, such as lactic acid and acetic acid [16]. On the other hand, the intestinal microbiome of formula-fed infants has a lower occupancy of *Bifidobacteria* than breast-fed infants [17–19]. Wang *et al*. [20] showed that soaped PA, which is more excreted in formula-fed infants than breast-fed infants [9–12], inhibits the growth of *Bifidobacteria* but not *Lactobacillae*. Therefore, formula-fed infants may face difficulties acquiring low intestinal pH due to inhibited *Bifidobacteria* growth. Moreover, soaped PA is an alkaline salt; hence, it is expected that soaped PA has a direct effect on increasing intestinal pH. Our previous study showed that higher fecal pH was associated with formula-feeding [21].

One of the strategies to achieve similar intestinal environments between formula-fed and breast-fed infants is fortifying the sn-2 palmitate. Several studies on the high sn-2 palmitate (approximately 40% of binding ratio to the sn-2 position) have shown that the fecal soaped PA of sn-2 palmitate fortified formula-fed infants decreased more than that of control formula-fed infants [9–13]. Our research team also reported that newborns fed formula with over 50% of PA bound to sn-2 are expected to have similar levels of fecal soaped and/or total PA levels to breast-fed infants [21]. Furthermore, several studies have shown that fortifying the sn-2 palmitate increased the *Bifidobacteria* levels in exclusively formula-fed infants [22,23]. Therefore, fortifying the sn-2 binding ratio of PA in infant formula can improve the intestinal environment of formula-fed infants, via elevated growth of *Bifidobacteria* and lower intestinal pH.

However, to the best of our knowledge, no studies have evaluated the intestinal environmental changes related to increased soaped PA levels using clinical data. This study was conducted as a secondary analysis of our previous observational study from Japan [21] to evaluate the associations between soaped PA levels and growth inhibition of intestinal microbiome, and to evaluate changes in the intestinal environment of formula-fed infant.

## Materials and methods

### Categorization of infant formula in Japan

The categorization of infant formula followed a previous report [21]. In Japan, there are two commercially available formulas with 50% or more of PA bound to the sn-2 position. We categorized these products as high sn-2 formula and the others as low sn-2 formula (Table 1). S1 Table shows the composition of fat and the ratio of PA to total fat in each formula and breast milk.

**Table 1:** Categorization of infant formula in Japan based on the ratio of sn-2 PA to total PA.

Infant formula	Percentage of sn-2 PA to total PA	Category
A	55.3	High sn-2 formula
B	52.9	
C	39.5	Low sn-2 formula
D	37.5	
E	11.9	
F	11.7	
G	11.4	
H	10.3	
I	6.4	
J	6.1	

### Study design and participants

This study is a secondary analysis of our previous multicenter observational study from Tokyo, Japan [21]. Clinical visits were scheduled at 1 month old. All legal guardians of each infant provided written informed consent before participating in the study, which was conducted in accordance with the Declaration of Helsinki. The study protocol was approved by the Ethics Committee of Juntendo University Hospital (approval numbers: 17–313, 20–333), the Research Ethics Committee of the University of Tokyo (approval numbers: 2018011NI, 2020354NI), and the ethics committee of Toho University Omori Medical Center (approval numbers: M18030, M20327). The inclusion criteria have been described previously [21]. Infant-mother pairs who provided informed consent were enrolled in the following terms: between September 1, 2018 – March 31, 2019, and between January 7, 2021 – June 30, 2022 in Juntendo University Hospital; between November 28, 2018 – September 30, 2019, and between February 26, 2021 – June 30, 2022 in The University of Tokyo Hospital; between September 1, 2018 – September 30, 2019, and between May 1, 2021–June 30, 2022 in Toho University Omori Medical Center.

### Data and sample collection

Demographic, feeding, and stool biochemistry (pH, soaped PA levels) data were collected from the previous study [21].

For the microbiome analysis, feces were collected by rectal swab sampling at the 1-month-clinical visit. The biological samples were stored in DNA/RNA shield solution (Zymo Research), and at –80 °C until analysis.

### Microbiome analysis

The microbial DNA from feces was extracted by ZymoBIOMICS™ DNA Miniprep Kit (Zymo Research) according to the manufacturer's protocol. The frozen samples were thawed at room temperature.

The extracted DNA was quantified using the Quant-iT™ dsDNA Assay Kits, high sensitivity (Thermo Fisher Scientific), or Qubit^TM^ dsDNA HS Assay Kit (Thermo Fisher Scientific). The V1-V2 region was amplified and purified by the Quick-16S NGS Library Prep Kit (Zymo Research). The final library of amplicon was obtained by mixing equal amounts of each sample’s library. For DNA sequencing, the Miseq Illumina sequencing platform (Illumina, USA) was performed using a 500 cycles v2 sequencing kit (2 × 250 bp paired-end reads; Illumina) or MiSeq reagent kit v3 (2 × 300 bp paired-end reads; Illumina). The library concentration during sequencing was 8 pmol/L, and 20% PhiX DNA was added at the same concentration. High-quality clean tags were clustered into operational classification units (OTUs) using USEARCH based on 97% sequence similarity using QIIME. Representative OTUs were used for further analysis using the SILVA bacterial database with the RDP algorithm. Data are reported as the relative abundance of *Bifidobacteria* and *Lactobacillae*.

### Statistical analysis

To evaluate the association between the feeding volume of high or low sn-2 formula and *Bifidobacteria* or *Lactobacillae* occupancy in the total microbiome at 1-month, multiple regression analysis using the feeding volume of high and low sn-2 formula as explanatory variables was conducted. The feeding volume was calculated as the average feeding volume per weight during the week before the 1-month check-up. We evaluated this association based on partial regression coefficients (β) and 95% confidential intervals (95%CIs) of feeding volume of high and low sn-2 formula. The use of antibiotics in infants and mothers, parity, gestational age at birth, *Bifidobacterium* supplementation of mothers (only for the model of *Bifidobacteria*), *Lactobacillus* supplementation of mothers (only for the model of *Lactobacillae*), and C-section birth were considered as confounders [24–28].

To evaluate the association between fecal soaped PA levels and the *Bifidobacteria* or *Lactobacillae* occupancy in the total microbiome at 1-month, multiple regression analysis using fecal soaped PA levels as explanatory variables was conducted. We evaluated this association based on the β and 95%CI of fecal soaped PA levels. In each analysis model, the same confounders as mentioned in the previous paragraph were considered.

**Fig 1:**
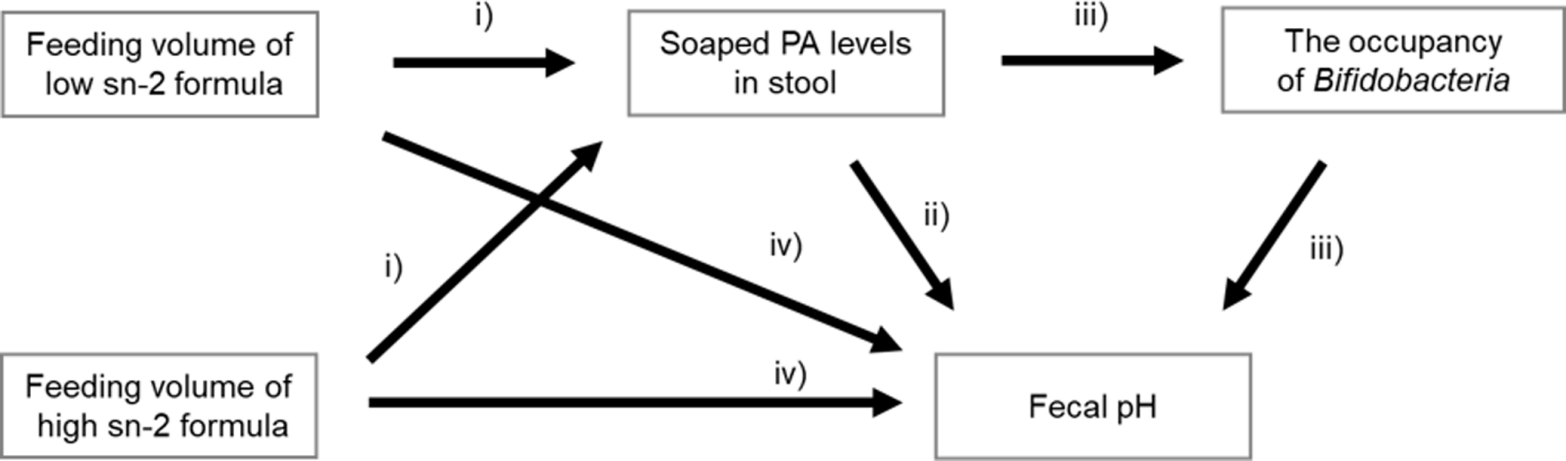
The hypothesized path model. The model was constructed according to the following elements: i) fecal soaped PA levels are lower in sn-2 palmitate fortified formula-fed than non-fortified formula-fed [10–12], ii) soaped PA is an alkaline salt, iii) soaped PA inhibits the growth of *Bifidobacteria* [20], which is known to produce acidic metabolite and thus reduces fecal pH, and iv) unknown path to increase stool pH by formula feeding. PA, palmitic acid.

A series of associations between soaped PA levels in stools, *Bifidobacteria* occupancy, and stool pH by feeding high or low sn-2 formula were investigated by path analysis. Fig 1 shows the hypothesized path model of the changes in intestinal factors (fecal soaped PA levels, *Bifidobacteria* occupancy, and fecal pH) with formula feeding. The p-value, comparative fit index (CFI), Tucker-Lewis index (TLI), root mean square error of approximation (RMSEA), and standardized root mean square residual (SRMR) were used to evaluate the goodness of model fit. Generally, p-values of ≥ 0.05, a CFI of ≥ 0.95, a TLI of ≥ 0.95, an RMSEA of ≤ 0.06, and an SRMR of ≤ 0.08 were considered a criteria for a goodness of model fit [29].

Mother-infant pairs with missing data were excluded from each statistical analysis. Statistical significance was set at a p-value of < 0.05. In multiple regression models, the 95%CI of β not crossing 0 was considered statistically significant. All statistical analyses were performed using the R statistical software (version 4.1.3) and BellCurve for Excel (version 3.20).

## Results

### Demographic data

As this study was conducted as a secondary analysis to focus on the intestine environmental factors, the demographic data was obtained from our previous report [21]. The flow chart of participants and demographic data are shown in S1 Fig and S2 Table, respectively.

### Microbiome analysis

**Fig 2:**
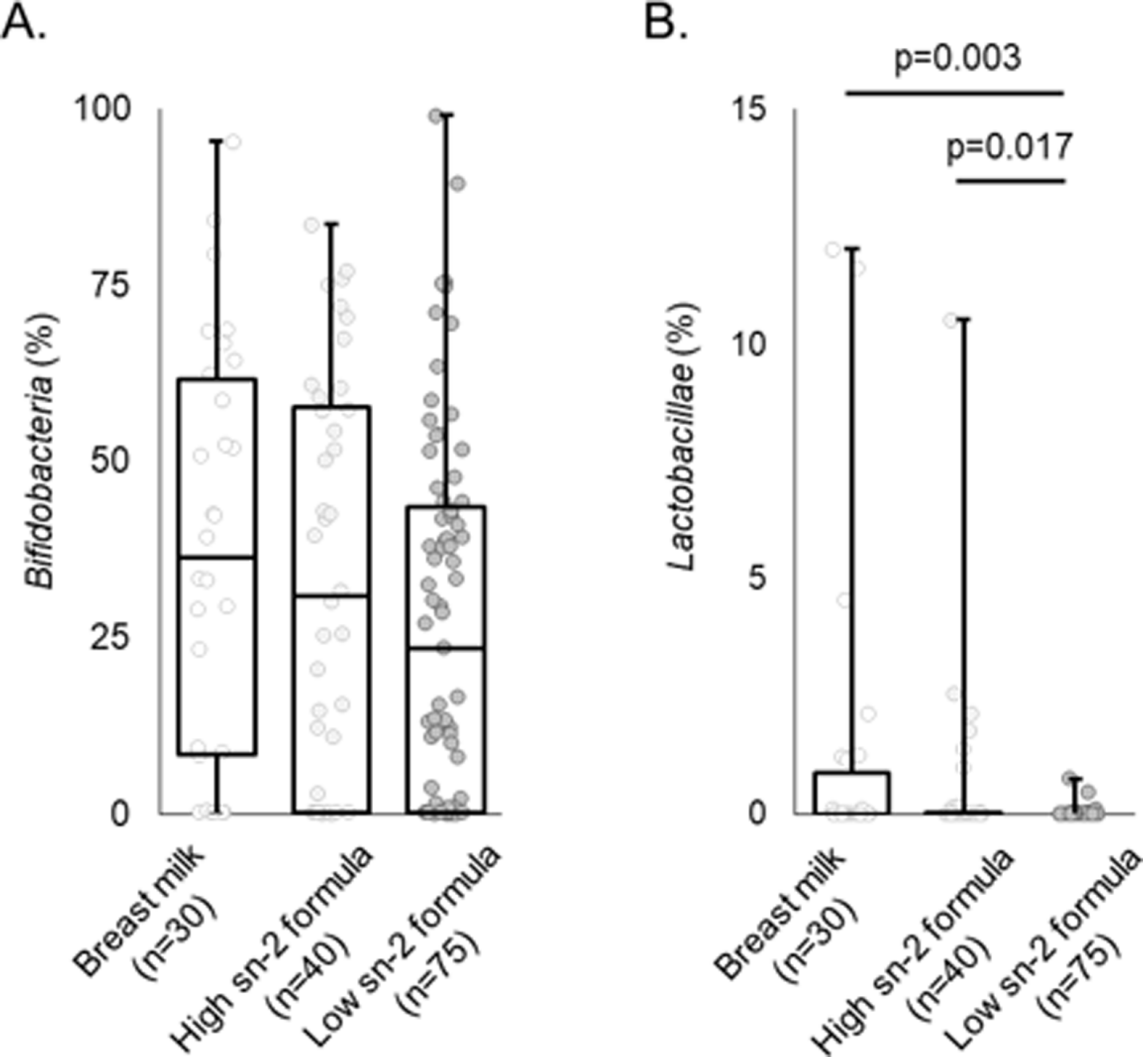
Results of the occupancy of *Bifidobacteria* and *Lactobacillae* in the intestinal microbiome of infants at 1 month of age. “Breast milk” represents those who were not recorded as formula-fed infants. “High or Low sn-2 formula” represents those who were recorded the feeding of high or low sn-2 formula at least once. All feeding types were determined using data during the week before the checkup at 1 month old. Steel-Dwass analysis was performed for the statistical test. PA, palmitic acid.

Fig 2 shows the results of the analysis of *Bifidobacteria* and *Lactobacillae* occupancy in the intestinal bacteria at 1 month of age. There was no significant difference in *Bifidobacteria* occupancy among breast-fed, high sn-2 formula-fed, and low sn-2 formula-fed infants. In contrast, the *Lactobacillae* occupancy of the low sn-2 formula-fed infants was significantly lower than that of the breast-fed and high sn-2 formula-fed infants. These indicators of intestinal bacteria in infants were used in the subsequent analysis.

### Associations between feeding volume of high or low sn-2 formula and *Bifidobacteria* or *Lactobacillae* occupancy in infants at 1 month of age

**Fig 3:**
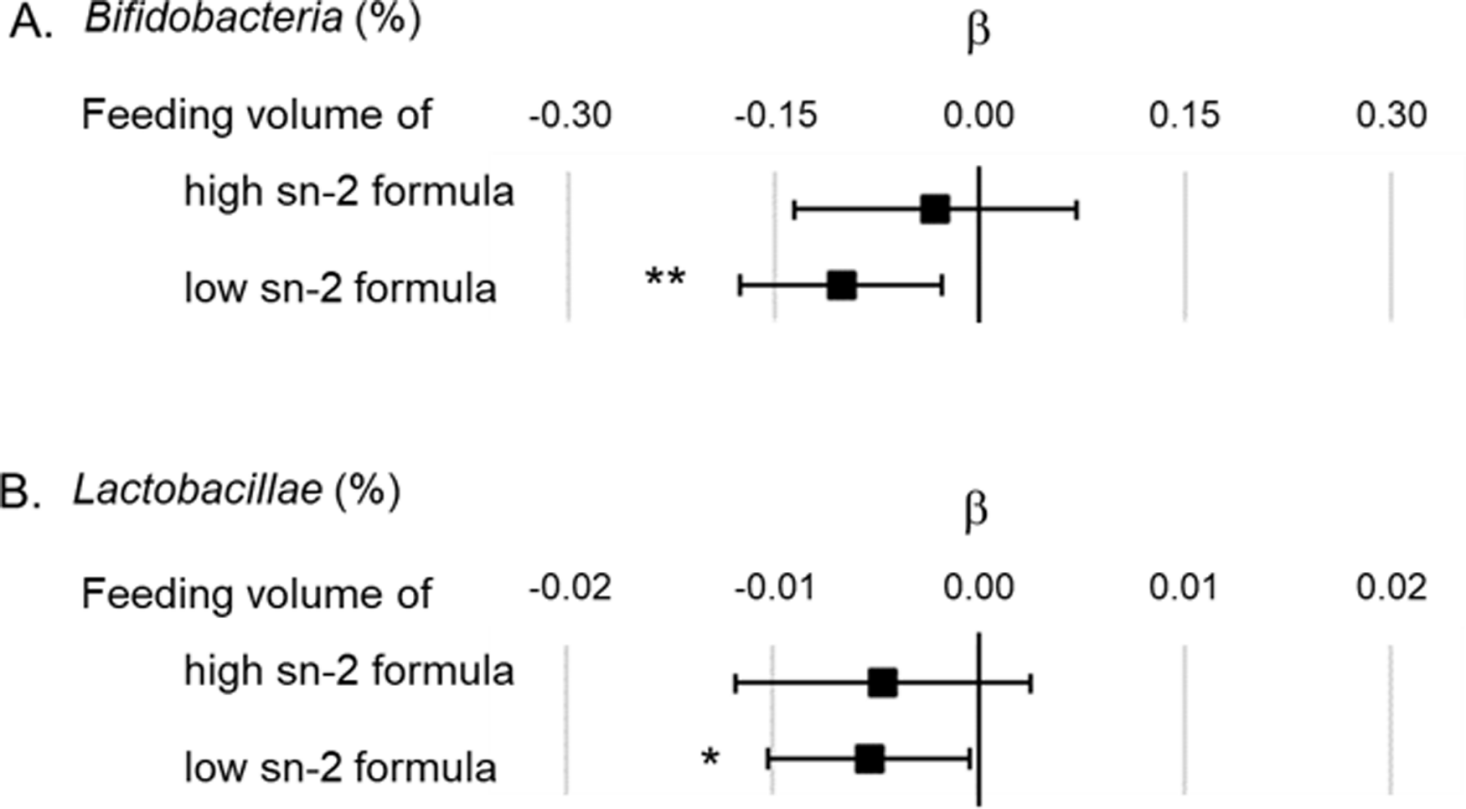
Associations between feeding volume of high or low sn-2 formula and *Bifidobacteria* or *Lactobacillae* occupancy in the multiple regression models. **A.** Association between feeding volume of high or low sn-2 formula and the *Bifidobacteria* occupancy in the total microbiome. **B.** Association between feeding volume of high or low sn-2 formula and the *Lactobacillae* occupancy in the total microbiome. Each model was adjusted for use of antibiotics in infants, use of antibiotics in mothers, parity, gestational age at birth, *Bifidobacterium* supplementation of mothers (only for the model of *Bifidobacteria*), *Lactobacillus* supplementation of mothers (only for the model of *Lactobacillae*), and C-section birth. The unit for feeding volumes indicates mL/day/kg. β indicates the partial regression coefficient. ■ indicates the partial regression coefficient of each factor, and the bar indicates the 95% confidence interval of β. * p < 0.05, ** p < 0.01. PA, palmitic acid.

Multiple regression analysis was performed to evaluate the associations between feeding volumes of high or low sn-2 formula and *Bifidobacteria* or *Lactobacillae* occupancy at 1 month of age. The results illustrated that both *Bifidobacteria* and *Lactobacillae* occupancy showed negative but no significant association with feeding volume of high sn-2 formula (β = −0.03, 95% CI = −0.14–0.07 [*Bifidobacteria* occupancy]; β = −0.005, 95% CI = −0.012–0.002 [*Lactobacillae* occupancy]), while a negative and significant association was observed with low sn-2 formula feeding volume (β = −0.10, 95% CI = −0.17– − 0.03 [*Bifidobacteria* occupancy]; β = −0.0053, 95% CI = −0.0102–0.0004 [*Lactobacillae* occupancy]) (Fig 3). The β and 95% CI of all explanatory variables in the multiple regression models are shown in S3 and S4 Tables.

### Associations between fecal soaped PA levels and *Bifidobacteria* or *Lactobacillae* occupancy in infants at 1 month of age

**Fig 4:**
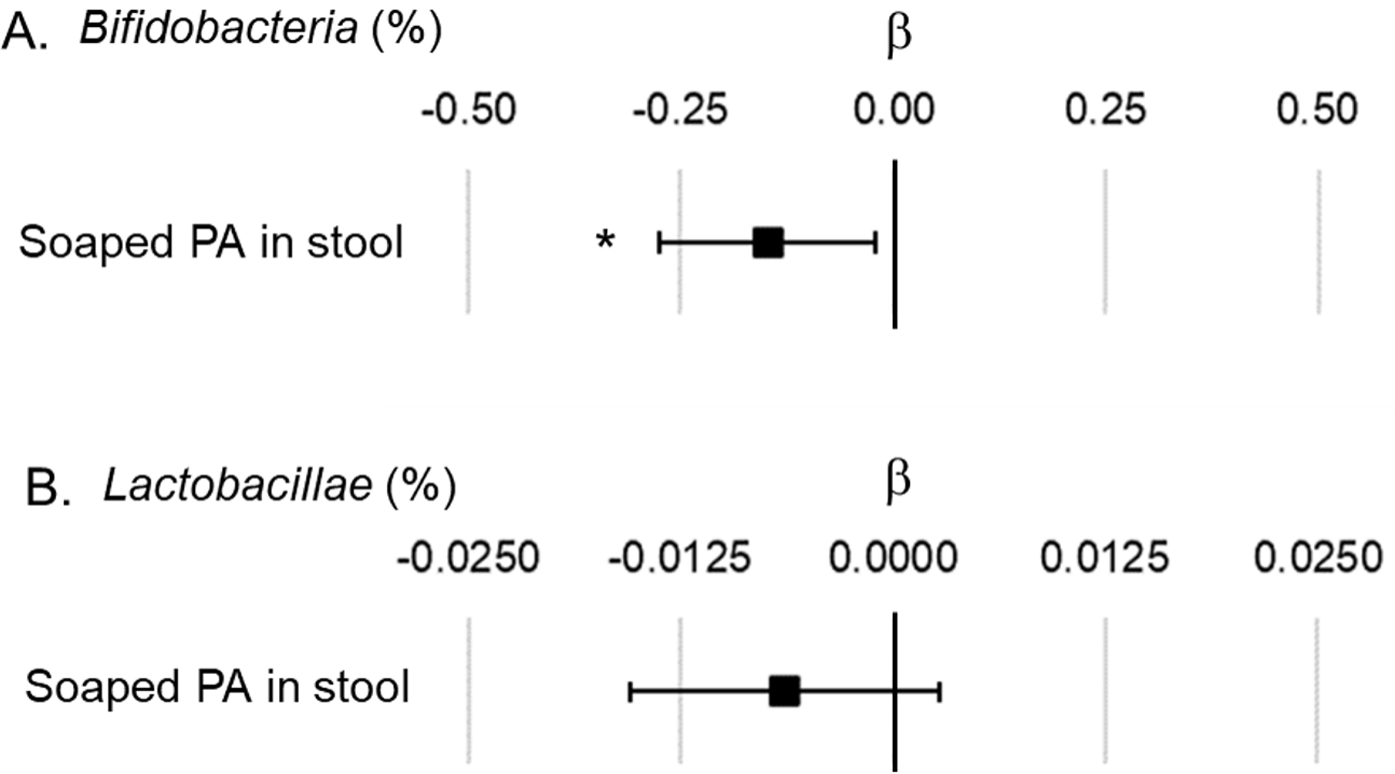
Associations between fecal soaped PA levels in the multiple regression models showing *Bifidobacteria* or *Lactobacillae* occupancy in infants. A. Association between fecal soaped PA levels and the *Bifidobacteria* occupancy in the total microbiome. B. Association between fecal soaped PA levels and the *Lactobacillae* occupancy in the total microbiome. Each model was adjusted for antibiotic use in infants, antibiotic use in mothers, parity, gestational age at birth, *Bifidobacterium* supplementation of mothers (only for the model of *Bifidobacteria*), *Lactobacillus* supplementation of mothers (only for the model of *Lactobacillae*), and C-section birth. The unit for fecal soaped PA levels indicates mg/g-dry-stool. β indicates the partial regression coefficient. ■ indicates the partial regression coefficient of each factor, and the bar indicates the 95% confidence interval of β. * p < 0.05. PA, palmitic acid.

Fig 4 shows the results of the multiple regression analysis to evaluate the association between fecal soaped PA levels and *Bifidobacteria* or *Lactobacillae* occupancy in infants at 1 month. The *Bifidobacteria* occupancy showed a negative correlation with fecal soaped PA levels (β = −0.15, 95% CI = −0.28– − 0.02), while *Lactobacillae* occupancy showed no significant association with fecal soaped PA levels (β = −0.006, 95% CI = −0.015–0.003). The β and 95% CI of all explanatory variables in the multiple regression models are shown in S5 and S6 Tables.

### Path analysis to evaluate the changes in the intestinal environment of newborns by formula-feeding

**Fig 5:**
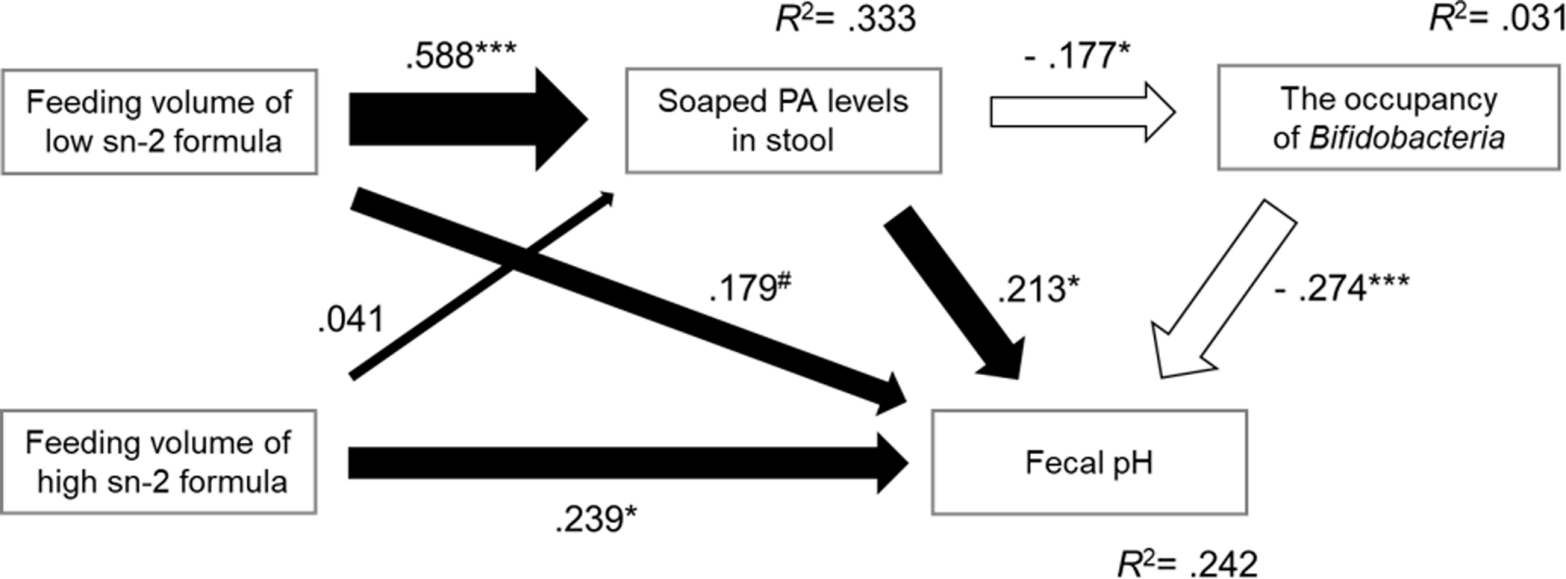
Standardized coefficients in the path model between feeding volume of high or low sn-2 PA formula and intestinal environmental factors. The numbers on the arrows indicate path coefficients. Black arrows indicate positive associations, and white arrows indicate negative associations. ^#^ p < 0.1, * p < 0.05, ** p < 0.01, *** p < 0.001. PA, palmitic acid.

Fig 5 shows the results of evaluating the hypothesized path model. None of the indicators of model fit were rejected, indicating that the model was valid (p = 0.262, CFI = 0.993, TLI = 0.970, RMSEA = 0.052 [90%CI = 0.000–0.209], SRMR = 0.029). Results of the path analysis showed a significant increase in the fecal soaped PA levels in the association between the feeding volume only in low sn-2 formula. Moreover, an increase in fecal soaped PA levels was also associated with an increase in fecal pH. Partial mediation by a decrease in *Bifidobacteria* occupancy was shown in the association between fecal soaped PA levels and increased fecal pH. The feeding volume of high sn-2 formula fed was not associated with an increase in fecal soaped PA levels, showing no difference between the intestinal environment and the feeding volume of high sn-2 formula. In contrast, the feeding volume of high sn-2 formula was associated with an increase in fecal pH that was not mediated by the increase in fecal soaped PA levels.

## Discussion

The present observational study suggests that increasing fecal soaped PA levels inhibit the growth of the intestinal microbiome using clinical data. Additionally, the path analysis demonstrated a mechanism which potentially explains how low sn-2 formula feeding causes the changes in the newborn’s intestinal tract: i) feeding low sn-2 formula increased fecal soaped PA levels, ii) these increased soaped PA levels inhibited the growth of *Bifidobacteria*, and iii) the association between fecal soaped PA levels and fecal pH is partially mediated by decreasing *Bifidobacteria* levels. On the other hand, feeding high sn-2 formula was not associated with *Bifidobacteria* levels, in addition to with fecal soaped PA levels, which is reported in our previous study [21]. Thus, the present study indicates that feeding with high sn-2 formula reduced the changes in intestinal environments associated with fecal soaped PA levels. However, it should be noted that high sn-2 formula feeding also increased the fecal pH, through a mechanism other than an increasing fecal soaped PA levels. Yaron *et al*. [22] and Wu *et al*. [23] have independently reported in their study that exclusive use of feeding the sn-2 palmitate fortified formula decreases fecal soaped PA levels and enhances growth of *Bifidobacteria*. These studies evaluated the effect of sn-2 palmitate on gut *Bifidobacteria* levels by comparison between groups using randomized controlled trials and did not evaluate the dose-dependent effect. Thus, a novel finding of this study is that a dose-dependent association between sn-1 or -3 palmitate intake and intestinal environmental factors was observed. Furthermore, the association between fecal soaped PA levels and *Bifidobacteria* growth has never been analyzed in the clinical data of newborns. This is the first study to demonstrate changes in the intestinal environment of formula-fed newborns with different PA sn-2 binding ratios of infant formulas.

Our results suggest that soaped PA is a factor that inhibits the formation of *Bifidobacteria*-enriched intestinal microbiome. The *in vitro* study by Wang *et al*. [20] showed that the cell wall of *Faecalibacterium prausnitzii* was thinned by soaped PA. Moreover, they also showed that the function of the surviving bacteria also decreases. They also reported that soaped PA inhibits the growth of *Bifidobacteria* and concluded that soaped palmitate inhibits the *in vitro* growth of some gut commensals by damaging the structure and function of their cell envelope. Our clinical data also showed that increasing fecal soaped PA levels was associated with decreasing the *Bifidobacteria* levels (Fig 4A). On the other hand, no significant association was observed between the fecal soaped PA levels and *Lactobacillae* levels (Fig 4B). Wang *et al*. [20] did not also observe the inhibition of *Lactobacillae* by soaped PA, similar to our results from clinical data. It is unknown why the growth of *Lactobacillae* was not affected by soaped PA in the report by Wang *et al*. [20] or in our clinical study. Preventing the increase in fecal soaped PA levels by infant formula with high sn-2 palmitate is essential for the formation of a breast-fed infants-like intestinal microbiome.

An increase in fecal soaped PA levels not only inhibits the growth of *Bifidobacteria,* but also reduces the acidity of the intestine. *Bifidobacteria* are known to produce acidic metabolites such as short-chain fatty acids, including lactic and acetic acids, by consuming oligosaccharides in breast milk [16,30–32]. The lower intestinal pH has shown to inhibit the growth pathogenic bacteria [33,34]. Breast milk, which is rich in prebiotics, is known to help the development of an acidic environment in the intestine by promoting the growth of *Bifidobacteria*. The results of the path analysis in this study showed that the fecal soaped PA levels lowered *Bifidobacteria* levels in the intestine, and finally led to higher fecal pH (Fig 5). Therefore, preventing higher fecal soaped PA levels in formula-fed infants is essential to develop an acidic intestinal environment similar to breast-fed infants.

The present study also showed that increasing the sn-2 binding ratio of PA alone is not sufficient to prevent higher intestinal pH in formula-fed infants. High sn-2 formula feeding was neither associated with the increase in fecal soaped PA levels nor with decreased *Bifidobacteria* occupancy unlike low sn-2 formula feeding (Figs 3A and 5), suggesting that high sn-2 formula feeding provides similar intestinal environments to breastfeeding. However, the positive association between high sn-2 formula feeding and fecal pH was shown through a different path other than increasing soaped PA levels (Fig 5). It is expected that another factor is required to develop lower intestinal pH, such as high amounts of HMOs. While HMOs are the third most significant component of breast milk [35], few HMOs and prebiotic components are contained in infant formulas [36]. HMOs reach the large intestine without digestion and serving as prebiotic nutrients [37]. Both factors, which are a high HMOs content to promote *Bifidobacteria* growth, in addition to a high sn-2 palmitate to remove factors that inhibit *Bifidobacteria* growth, are probably essential for further promoting an intestinal environment similar to that of breast-fed infants. Yao *et al*. [10] and Nowacki *et al*. [11] reported that *Bifidobacteria* levels increase with the combination of sn-2 palmitate and prebiotics. Although fecal pH was not measured in their studies, it is expected to decrease fecal pH corresponding to higher *Bifidobacterial* growth. Other than this, there have been no reports on the effect of the combination of sn-2 palmitate and HMOs on intestinal environments. Thus, further modifications in the types and amounts of prebiotics may be necessary to achieve lower intestinal pH. The results of this study lead us to hypothesis that a high sn-2 formula successfully eliminates factors that inhibit *Bifidobacteria* growth levels, although fecal pH does not decrease as much as expected due to lack of components to support *Bifidobacteria* growth.

This study has few limitations. First, this was an observational study; hence, causal associations could not be demonstrated. Second, the components of the formulas other than the ratio of PA bound to the sn-2 position were not standardized. Therefore, factors other than the PA sn-2 binding ratio as latent factors mediating the associations observed in this study cannot be ruled out. Third, the analysis did not consider variations in breast milk components between individuals. We assumed that the PA sn-2 binding ratio of breast milk is high and constant in the evaluation. Indeed, we confirmed that the sn-2 binding ratio of PA in the breast milk of the participants in this study was approximately 70% [21]. Fourth, the feeding volume of breast milk was not obtained. Since the feeding volume of breast milk strongly inversely correlates with the feeding volume of infant formula, we decided not to add the feeding volume of breast milk as an explanatory variable in the multiple regression analysis model.

## Conclusions

The clinical data in this study support that soaped PA inhibits *Bifidobacteria* growth, which agrees with the report of an *in vitro* study. Furthermore, our clinical data from newborns showed that feeding infant formula with low sn-2 palmitate is associated with an increase in fecal soaped PA, which leads to a lower *Bifidobacteria* level, followed by an increase in fecal pH.

## Supporting information

S1 Table Palmitic acid (PA) ratio in sn-2 position, PA ratio in total fat, and fat and energy content in breast milk at 1 month and infant formula available in Japan^†^. ^†^ Referenced from a previous report [21].
(PDF)

S2 Table Demographic data. Demographic data was obtained from our previous cohort study [21].
(PDF)

S3 Table Associations between feeding volume of high sn-2 formula and *Bifidobacteria* occupancy in infants at 1 month of age in multiple regression analysis (all explanatory variables). (PDF)

S4 Table Associations between feeding volume of high sn-2 formula and *Lactobacillae* occupancy in infants at 1 month of age in multiple regression analysis (all explanatory variables). (PDF)

S5 Table Associations between fecal soaped PA levels and *Bifidobacteria* occupancy in infants at 1 month of age in multiple regression analysis (all explanatory variables). (PDF)

S6 Table Associations between fecal soaped PA levels and *Lactobacillae* occupancy in infants at 1 month of age in multiple regression analysis (all explanatory variables). (PDF)

S1 Fig Flow chart of participants. The data of participants was extracted from our previous cohort study [21].
(PDF)
