# Supplementary material for: Feeding infant formula with low sn-2 palmitate causes changes in newborn’s intestinal environments through an increase in fecal soaped palmitic acid
Source: PLoS One. 2025 May 28;20(5):e0324256. doi: 10.1371/journal.pone.0324256 (PMC12118907; doi:10.1371/journal.pone.0324256)
Supplement: S1 Table — † Referenced from a previous report [21]. (PDF) [file pone.0324256.s001.pdf]

S1 Table

S1 Table. Palmitic acid (PA) ratio in sn-2 position to total PA, PA ratio to total fat, and fat and energy content in breast milk at 1 month and infant formula available in Japan†

Sample		sn-2 PA to total PA<br>%	PA to total fat<br>%	Fat<br>g/100 mL	Protein<br>g/100 mL	Carbohydrate<br>g/100 mL	Energy<br>kcal/100 mL	Category
Breast milk		73.9 ± 6.1*1	22.6 ± 2.2*2	3.6 ± 1.3*3	1.5 ± 0.3*3	7.8 ± 0.8*3	69 ± 12*3	-
Infant formula	A	55.3	20.6	3.5	1.5	7.8	68	High sn-2 PA milk
	B	52.9	20.2	3.5	1.7	7.7	68	
	C	39.5	22.0	3.6	1.5	7.1	66	Low sn-2 PA milk
	D	37.5	21.8	3.8	1.4	7.1	68	
	E	11.9	19.3	3.6	1.4	7.3	67	
	F	11.7	25.3	3.6	1.5	7.4	67	
	G	11.4	19.2	3.6	1.5	7.2	67	
	H	10.3	18.2	3.6	1.4	7.3	67	
	I	6.4	23.3	3.5	1.4	7.5	67	
	J	6.1	22.9	3.5	1.4	7.5	67	

*1: n=139, *2: n=140, *3: n=92
† Referenced from a previous report [1].
The ratio of sn-2 PA and PA in total PA of infant formula and breast milk, the fat/protein/carbohydrate/energy levels in breast milk were analyzed in previous reports [1]. The fat/protein/carbohydrate/energy contents in infant formula were obtained from product information.

[1] Shoji H, Arai H, Kakiuchi S, Ito A, Sato K, Jinno S, et al. Infant formula with 50% or more of palmitic acid bound to the sn-2 position of triacylglycerols eliminate the association between formula-feeding and the increase of fecal palmitic acid levels in newborns: An exploratory study. Nutrients. 2024; 16: 1558.
