# Supplementary material for: Feeding infant formula with low sn-2 palmitate causes changes in newborn’s intestinal environments through an increase in fecal soaped palmitic acid
Source: PLoS One. 2025 May 28;20(5):e0324256. doi: 10.1371/journal.pone.0324256 (PMC12118907; doi:10.1371/journal.pone.0324256)
Supplement: S2 Table — Demographic data was obtained from our previous cohort study [21]. (PDF) [file pone.0324256.s002.pdf]

S2 Table

S2 Table. Demographic data

		Total (n=149)	Feeding type: Exclusively breast-fed infants (n=31)	Formula-fed infants: High sn-2 formula (n=40)	Formula-fed infants: Low sn-2 formula (n=78)	p value*
Mothers						
age at birth	years	34.4 ± 5.4	32.9 ± 4.8	34.5 ± 5.3	35.0 ± 5.5	0.172
BMI before pregnancy	-	21.5 ± 3.5	21.1 ± 3.0	21.5 ± 3.5	21.6 ± 3.7	0.816
maximum BMI during pregnancy	kg/m^2	25.5 ± 3.3	25.1 ± 2.7	25.6 ± 3.6	25.6 ± 3.4	0.749
nationality (not Japan)	person	2 (1.3%)	0 (0%)	1 (2.5%)	1 (1.3%)	0.667
missing		1 (0.7%)	1 (3.2%)	0 (0%)	0 (0%)	
Has given birth before	person	69 (46.3%)	15 (48.4%)	17 (42.5%)	37 (47.4%)	0.849
c-section	person	47 (31.5%)	8 (25.8%)	15 (35.0%)	25 (32.1%)	0.704
administration of antimicrobials before birth (Yes)	person	25 (16.8%)	6 (19.4%)	6 (15.0%)	13 (16.7%)	0.856
missing		1 (0.7%)	1 (3.2%)	0 (0%)	0 (0%)	
intake of *Lactobacillus*/*Bifidobacteria* supplements during pregnancy (Yes)	person	8 (5.4%)	1 (3.2%)	2 (5.0%)	5 (6.4%)	0.759
missing		1 (0.7%)	1 (3.2%)	0 (0%)	0 (0%)	
fathers						
nationality (not Japanese)	person	5 (3.4%)	1 (3.2%)	2 (5.0%)	2 (2.6%)	0.786
missing		1 (0.7%)	1 (3.2%)	0 (0%)	0 (0%)	
infants						
girls	person	80 (53.7%)	16 (51.6%)	24 (60.0%)	40 (51.3%)	0.665
missing		1 (0.7%)	1 (3.2%)	0 (0%)	0 (0%)	
gestational age at birth	week	39.3 ± 1.1	39.3 ± 0.9	39.3 ± 1.2	39.2 ± 1.2	0.928
weight at birth	g	3058.5 ± 304.9	3059.7 ± 280.9	3070.6 ± 295.1	3051.7 ± 321.7	0.951
length at birth	cm	48.7 ± 2.9	48.6 ± 1.5	48.6 ± 1.6	48.8 ± 3.8	0.907
head circumference at birth	cm	33.6 ± 1.2	33.4 ± 1.2	33.7 ± 1.1	33.7 ± 1.2	0.452
age at one-months checkup	day	33.6 ± 5.3	33.9 ± 5.6	33.9 ± 4.0	33.3 ± 5.7	0.790
use of antimicrobials until 1 month	person	3 (2.0%)	0 (0%)	0 (0%)	3 (3.8%)	0.248
feeding volume of infant formula	mL/day/kg	-	-	67.4 ± 57.3	84.7 ± 61.8	0.143

*: One-way analysis of variance or Kruskal-Wallis test or chi-square test
Demographic data was obtained from our previous cohort study [1].

[1] Shoji H, Arai H, Kakiuchi S, Ito A, Sato K, Jinno S, et al. Infant formula with 50% or more of palmitic acid bound to the sn-2 position of triacylglycerols eliminate the association between formula-feeding and the increase of fecal palmitic acid levels in newborns: An exploratory study. Nutrients. 2024; 16: 1558.
