# Supplementary material for: Feeding infant formula with low sn-2 palmitate causes changes in newborn’s intestinal environments through an increase in fecal soaped palmitic acid
Source: PLoS One. 2025 May 28;20(5):e0324256. doi: 10.1371/journal.pone.0324256 (PMC12118907; doi:10.1371/journal.pone.0324256)
Supplement: S3 Table — (PDF) [file pone.0324256.s003.pdf]

S3 Table

S3 Table. Associations between feeding volume of high/low sn-2 formula and *Bifidobacteria* occupancy in infants at 1 month of age in multiple regression analysis (all explanatory variables)

Explanatory variables	β	95%CI	p value
Feeding volume of high sn-2 formula, mL/day/kg	-0.03	-0.14 – 0.07	0.538
Feeding volume of low sn-2 formula, mL/day/kg	-0.10	-0.17 – -0.03**	0.007
Use of antibiotics in infants, yes	-4.87	-36.0 – 26.2	0.757
Use of antibiotics in mothers, yes	-9.82	-21.0 – 1.34	0.084
Parity, more than twice	11.5	2.54 – 20.4*	0.012
Gestational age at birth, weeks	-7.93	-12.2 – -3.69***	< 0.001
Bifidobacteria supplementation of mothers, yes	43.0	12.0 – 73.9**	0.007
C-section birth, yes	-11.9	-21.6 – -2.2*	0.017

*: $p < 0.05$, **: $p < 0.01$, ***: $p < 0.001$.
