# Supplementary material for: Feeding infant formula with low sn-2 palmitate causes changes in newborn’s intestinal environments through an increase in fecal soaped palmitic acid
Source: PLoS One. 2025 May 28;20(5):e0324256. doi: 10.1371/journal.pone.0324256 (PMC12118907; doi:10.1371/journal.pone.0324256)
Supplement: S4 Table — (PDF) [file pone.0324256.s004.pdf]

S4 Table

S4 Table. Associations between feeding volume of high/low sn-2 formula and *Lactobacillae* occupancy in infants at 1 month of age in multiple regression analysis (all explanatory variables)

Explanatory variables	β	95%CI	p value
Feeding volume of high sn-2 formula, mL/day/kg	-0.005	-0.012 – 0.002	0.200
Feeding volume of low sn-2 formula, mL/day/kg	-0.0053	-0.0102 – -0.0004*	0.035
Use of antibiotics in infants, yes	-0.51	-2.53 – 1.51	0.617
Use of antibiotics in mothers, yes	1.03	0.27 – 1.79**	0.008
Parity, more than twice	0.05	-0.56 – 0.65	0.878
Gestational age at birth, weeks	0.04	-0.24 – 0.33	0.761
Lactobacillus supplementation of mothers, yes	0.69	-0.86 – 2.24	0.380
C-section birth, yes	0.13	-0.53 – 0.79	0.701

*: $p < 0.05$, **: $p < 0.01$.
