# Supplementary material for: Feeding infant formula with low sn-2 palmitate causes changes in newborn’s intestinal environments through an increase in fecal soaped palmitic acid
Source: PLoS One. 2025 May 28;20(5):e0324256. doi: 10.1371/journal.pone.0324256 (PMC12118907; doi:10.1371/journal.pone.0324256)
Supplement: S5 Table — (PDF) [file pone.0324256.s005.pdf]

S5 Table

S5 Table. Associations between fecal soaped PA levels and *Bifidobacteria* occupancy in infants at 1 month of age in multiple regression analysis (all explanatory variables)

Explanatory variables	β	95%CI	p value
Fecal soaped PA levels, mg/g-dry-stool	-0.15	0.28 – -0.02*	0.023
Use of antibiotics in infants, yes	0.38	-39.4 – 40.2	0.985
Use of antibiotics in mothers, yes	-7.20	-20.9 – 6.50	0.300
Parity, more than twice	11.9	1.97 – 21.8*	0.019
Gestational age at birth, weeks	-7.00	-11.8 – -2.23**	0.004
Bifidobacteria supplementation of mothers, yes	33.4	0.79 – 66.1*	0.045
C-section birth, yes	-8.05	-19.3 – 3.18	0.158

*: $p < 0.05$, **: $p < 0.01$.
