# Supplementary material for: Feeding infant formula with low sn-2 palmitate causes changes in newborn’s intestinal environments through an increase in fecal soaped palmitic acid
Source: PLoS One. 2025 May 28;20(5):e0324256. doi: 10.1371/journal.pone.0324256 (PMC12118907; doi:10.1371/journal.pone.0324256)
Supplement: S6 Table — (PDF) [file pone.0324256.s006.pdf]

S6 Table

S6 Table. Associations between fecal soaped PA levels and *Lactobacillae* occupancy in infants at 1 month of age in multiple regression analysis (all explanatory variables)

Explanatory variables	β	95%CI	p value
Fecal soaped PA levels, mg/g-dry-stool	-0.006	-0.015 – 0.003	0.169
Use of antibiotics in infants, yes	-0.72	-3.30 – 1.86	0.582
Use of antibiotics in mothers, yes	1.57	-0.60 – 2.55**	0.002
Parity, more than twice	-0.001	-0.70 – 0.70	0.997
Gestational age at birth, weeks	0.08	-0.25 – 0.42	0.624
Lactobacillus supplementation of mothers, yes	0.51	-1.30 – 2.32	0.580
C-section birth, yes	0.22	-0.58 – 1.01	0.589

**: $p < 0.01$.
