# Supplementary material for: Feeding infant formula with low sn-2 palmitate causes changes in newborn’s intestinal environments through an increase in fecal soaped palmitic acid
Source: PLoS One. 2025 May 28;20(5):e0324256. doi: 10.1371/journal.pone.0324256 (PMC12118907; doi:10.1371/journal.pone.0324256)
Supplement: S1 Fig — The data of participants was extracted from our previous cohort study [21]. (PDF) [file pone.0324256.s007.pdf]

S1 Fig

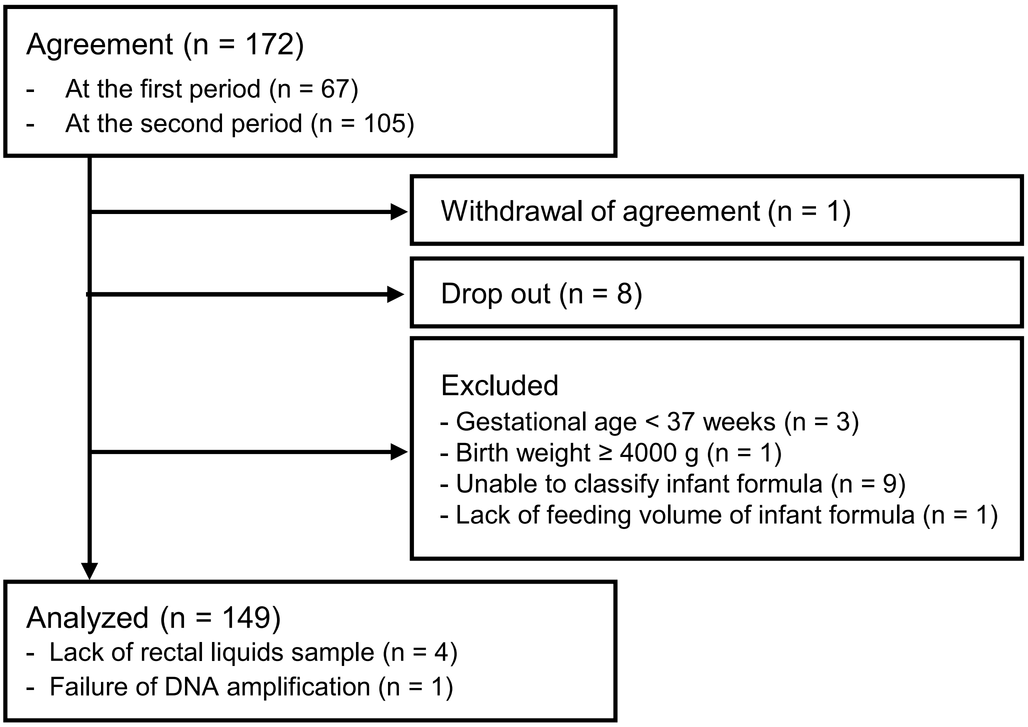

S1 Fig. Flow chart of participants.
The data of participants was extracted from our previous cohort study [1].

[1] Shoji H, Arai H, Kakiuchi S, Ito A, Sato K, Jinno S, et al. Infant formula with 50% or more of palmitic acid bound to the sn-2 position of triacylglycerols eliminate the association between formula-feeding and the increase of fecal palmitic acid levels in newborns: An exploratory study. Nutrients. 2024; 16: 1558.
